# Necrotizing Enterocolitis in a Patient With Newly Diagnosed Multiple Myeloma and Hyperviscosity Syndrome: A Case Report

**DOI:** 10.7759/cureus.41292

**Published:** 2023-07-02

**Authors:** Tanvi H Patel, Ramya Bachu, Ben Davis, Prashanth Damalcheruvu, Sharmilan Thanendrarajan

**Affiliations:** 1 Department of Internal Medicine, University of Arkansas for Medical Sciences, Little Rock, USA; 2 Department of Surgery, University of Arkansas for Medical Sciences, Little Rock, USA; 3 Department of Radiology, University of Arkansas for Medical Sciences, Little Rock, USA; 4 Department of Hematology and Oncology, University of Arkansas for Medical Sciences, Little Rock, USA

**Keywords:** necrosis, thrombosis, ischemia, hyperviscosity, multiple myeloma, necrotizing enterocolitis

## Abstract

Multiple myeloma (MM) is a clonal plasma cell proliferative disorder characterized by the abnormal increase of monoclonal paraprotein and can lead to specific end-organ damage. Necrotizing enterocolitis or bowel necrosis is a surgical emergency defined by cellular death because of reduced blood flow to the gastrointestinal tract. We report a case of a 75-year-old female who was diagnosed with hyperviscosity syndrome (HVS) and was sent to ED. Further workup showed that she had a new diagnosis of IgG kappa MM for which she was started on chemotherapy. Later, she developed respiratory distress and abdomen distention with less frequent bowel movements, and general surgery was consulted. CT scan of the abdomen and pelvis with contrast showed findings consistent with bowel ischemia vs infarction. The patient was immediately taken to the operating room, and exploratory laparotomy showed nonsurvivable bowel necrosis. She was transitioned to comfort care and passed away later. We aim to increase awareness among physicians to include HVS as one of the possible complications of MM and to detect it early to prevent morbidity and mortality.

## Introduction

Necrotizing enterocolitis is a surgical emergency defined by cellular death as a result of reduced blood flow to the gastrointestinal tract [1]. The most common reasons for bowel necrosis include acute arterial obstruction from thrombosis or embolism but it can also occur due to hyperviscosity syndrome (HVS) or venous thrombosis and symptoms can include abdominal pain, constipation, bloating, fever, nausea, and vomiting. HVS is a condition characterized by the thickening of the blood, which increases blood flow resistance [2]. HVS rarely develops in multiple myeloma (MM) but according to one study, incidence can be up to 4.2% among patients with IgG type [3]. HVS can be an oncologic emergency and treatment mainly involves addressing the underlying condition causing the thickening of the blood. Here, we describe a case of a patient who presented with HVS and was newly diagnosed with IgG kappa MM. She developed necrotizing enterocolitis and later passed away despite an emergency exploratory laparotomy. We believe that our patient represents a rare case of IgG MM associated with HVS and bowel necrosis. In such cases, early recognition and immediate treatment are essential to minimize the risk of complications and improve outcomes.

## Case presentation

A 75-year-old female with a past medical history of fibroadenoma presented to her primary care physician for back pain with radiculopathy in her right leg and fatigue that started approximately two months ago. She was diagnosed with pyriformis syndrome and was given a steroid injection that helped with her symptoms. She was also recently diagnosed with hypertension and was started on losartan. Routine blood work was attempted but was unsuccessful due to the blood being too thick. Multiple attempts were made to draw blood without success. The patient was referred to a hematologist for further workup. She was found to have hemoglobin of 6.9 g/dL (12-15 g/dL), IgG level of 11,577 mg/dL (700-1600 mg/dL), and viscosity >6.34 centipoise (1-1.5 centipoise). She also complained of 25 pounds of weight loss over the past couple of months, unsteady gait due to right leg pain, along with tingling and paresthesia in her right leg. She denied headache, blurry vision, dizziness, seizures, hearing problems, nose bleeds, chest pain, shortness of breath, nausea, vomiting, early satiety, abdomen pain, diarrhea, constipation, blood in stool, blood in urine, dysuria, recent falls, family history of blood cancers or coagulopathy. She was referred to our ED for further evaluation.

On arrival at the ED, she was afebrile, with blood pressure (BP) of 218/110 mm Hg, heart rate (HR) of 93/minute, respiratory rate of 16/minute, and oxygen saturation of 100% on room air. Laboratory test results (Table 1) showed hemoglobin 7.0 g/dL (12-15 g/dL), sodium 126 mmol/L (135-145 mmol/L), creatinine 1.1 mg/dL (0.4-1.0 mg/dL), and anion gap <1 (3-11). She was given losartan and nifedipine in ED, started on IV fluids, and admitted for further management. Because of elevated levels of IgG and viscosity, she underwent further workup for possible MM and she was found to have beta-2 microglobulin (B2M) 10.9 mg/L (1.1-2.5 mg/L), kappa-free light chain (KFLC) 96.52 mg/dL (0.33-1.94 mg/dL), lambda free light chain (LFLC) 0.17 mg/dL (0.57-2.63 mg/dL), K/L ratio 567.76 (0.26-1.65), IgG 8727 mg/dL (700-1600 mg/dL), IgA (immunoglobulin A) <20 mg/dL( 70-400 mg/dL), IgM (immunoglobulin M) <13 mg/dL (40-230 mg/dL), plasma viscosity 2.5 centipoise (repeat level 3.3 centipoise the following day, normal range 1.0-1.5 centipoise), total protein 13 g/dL (6.4-8.3 g/dL), albumin 2.3 g/dL (3.5-5.0 g/dL), calcium 8.1 mg/dL (8.6-10.2 mg/dL). Serum immunofixation showed IgG kappa plus free kappa M proteins.

**Table 1:** Laboratory test results at our institution B2M: beta-2 microglobulin; IgG: immunoglobulin G; IgA: immunoglobulin A; IgM: immunoglobulin M; KFLC: kappa free light chain; LFLC: lambda free light chain

Test	Results	Reference range
Hemoglobin	7 g/dL	12-15 g/dL
Sodium	126 mmol/L	135-145 mmol/L
Calcium	8.1 mg/dL	8.6-10.2 mg/dL
Anion gap	<1	3-11
Creatinine	1.1 mg/dL	0.4-1.0 mg/dL
Total protein	13 g/dL	6.4-8.3 g/dL
Albumin	2.3 g/dL	3.5-5.0 g/dL
Viscosity	2.5 centipoise	1.0-1.5 centipoise
B2M	10.9 mg/L	1.1-2.5 mg/L
IgG	8727 mg/dL	700-1600 mg/dL
IgA	<20 mg/dL	70-400 mg/dL
IgM	<13 mg/dL	40-230 mg/dL
KFLC	96.52 mg/dL	0.33- 1.94 mg/dL
LFLC	0.17 mg/dL	0.57-2.63 mg/dL
K/L ratio	567.76	0.26-1.65

Positron emission tomography (PET)/CT didn’t show dominant osteolytic lesions or extramedullary disease. MRI didn’t show focal lesions either but showed subacute sciatic nerve denervation changes. Bone marrow biopsy showed 70% plasma cells, flow cytometry showed 14.776% myeloma plasma cells. She was started on Velcade, dexamethasone, cisplatin, doxorubicin, cyclophosphamide, and etoposide for newly diagnosed IgG kappa MM. 

On the fourth day after admission, the patient started having mild bilateral leg swelling, and IV fluid was stopped; she was given furosemide 40 mg IV. Her creatinine improved from 1.1 on arrival to 0.9 but gradually increased to 1.4. Losartan was stopped in a setting of acute kidney injury (AKI), nifedipine was continued, and her BP improved significantly. On the fifth day after admission, the patient was found to be in mild respiratory distress along with mild tachycardia, although oxygen saturation was 95% on room air. The rapid response team was called, she was in atrial fibrillation (Afib) with rapid ventricular response (RVR). She was given IV furosemide 80 mg along with IV metoprolol 2.5 mg. Her HR came down and her breathing improved.

A chest X-ray didn’t show any acute findings, and CT pulmonary angiography (CTPA) for pulmonary embolus (PE) was not done in the setting of acute kidney injury (AKI). Ultrasound Doppler of the leg didn’t show deep venous thrombosis. The cause of respiratory distress was thought to be likely fluid overload after ruling out other common causes. The patient also improved after receiving furosemide. The patient was found to have urinary tract infection upon workup and was started on cefepime. 

**Figure 1:**
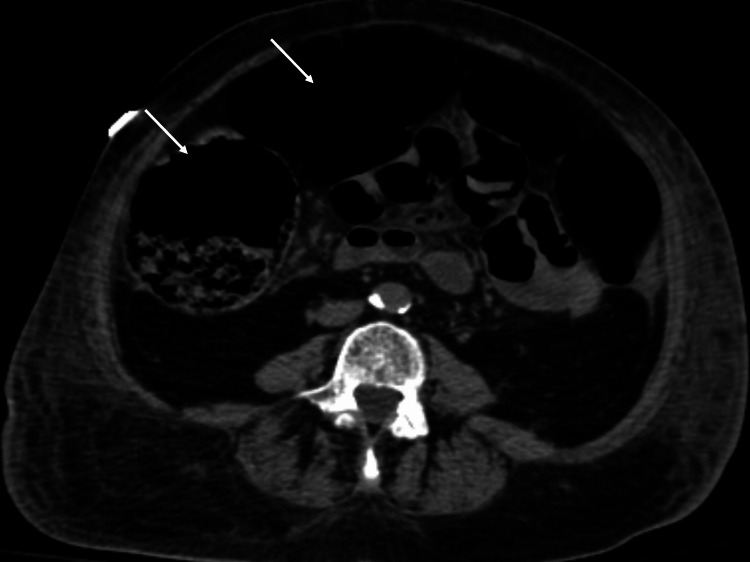
Initial CT scan of the abdomen pelvis without contrast showed non-specific mild distention of the colon (arrows). No finding of small bowel ischemia such as pneumatosis intestinalis, dilated small bowel loops, or portal venous air was noted

Approximately four to five days after admission, she was observed to have less frequent bowel movements along with mild abdomen distention. She was also noted to have low urine output; a Foley catheter was placed and she had good urine output after that. She continued to have abdomen distention with less frequent bowel movements and decreased gas production, and general surgery was consulted. CT scan of the abdomen pelvis without contrast showed nonspecific significant colon distention, but no signs of free abdominal air (Figure 1). General surgery recommended an aggressive bowel regimen; she was given bisacodyl suppository and enema, oral lactulose but still didn’t have bowel movement. The next day, i.e., day six after admission, she was found to have increased work of breathing again with worsening abdomen distention.

**Figure 2:**
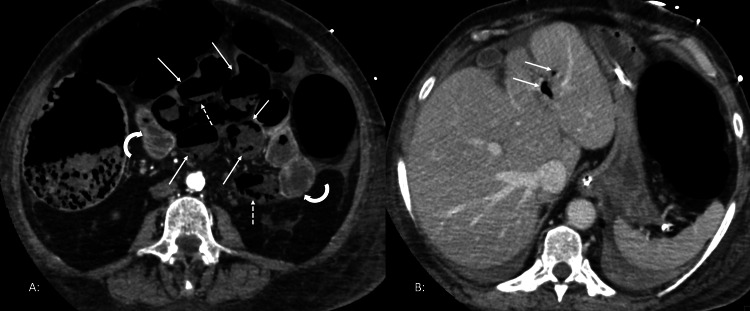
CT scans of the abdomen and pelvis with contrast showing findings of small bowel ischemia. (A) Multiple dilated loops of small bowel in the central abdomen, non-enhancement of the bowel wall (solid arrows) compared to normally enhancing adjacent bowel loops (curved arrows). There is also evidence of air in the bowel wall or pneumatosis intestinalis (dashed arrows); (B) Air within the portal venous radicals in the left hepatic lobe (solid arrows)

The patient was admitted to the medical intensive care unit (MICU) on the seventh day after admission as she needed intubation for acute hypoxic respiratory failure. The reason for respiratory failure was thought to be likely volume overload/low lung volumes due to worsening abdomen distention. CT scan of the abdomen pelvis with contrast was ordered, which showed multiple dilated loops of the small and large bowel with evidence of pneumatosis, minimal to no enhancement within several distal small bowel loops; the findings were consistent with bowel ischemia vs infarction (Figure 2). The patient was immediately taken to the operating room and an exploratory laparotomy was done. She was found to have nonsurvivable bowel necrosis. After a discussion with the family, she was transitioned to comfort care, and she passed away later that day.

## Discussion

Patients with MM often develop complications related to their disease, including hypercalcemia, skeletal lesions, infections, and acute or chronic renal insufficiency. Less common complications include hyperuricemia, neurological involvement, or HVS, which is a condition characterized by thickening of the blood and results in increased resistance to blood flow [4,5]. 

Hyperviscosity is defined based on the relative viscosity of serum as compared to water. When normal relative serum viscosity is 1.8 centipoise, symptoms of hyperviscosity develop at a level of 5 to 6, a level usually reached at paraprotein concentrations of approximately 7000 mg/dL for IgA, 5000 mg/dL for IgG, and 4000 mg/dL for IgM [2]. 

HVS develops rarely in MM but according to one study report, incidence can be up to 4.2% among patients with IgG type [3]. It could be due to the presence of high molecular weight polymers of IgG proteins in the serum. In our patient, the serum viscosity is greater than 6.34 centipoise, and a IgG level of 11, 577 mg/dL. 

The severity of symptoms is directly related to increased levels of serum viscosity [4]. It also results in increased circulating proteins, affecting platelet aggregation and causing prolonged bleeding time. The clinical triad of HVS includes mucosal bleeding, neurological deficits, and visual disturbances [6]. HVS is an oncologic emergency that, if not managed appropriately, could lead to multi-organ failure, including AKI, cardiac failure, pulmonary edema, myocardial infarction, stroke, etc. Intestinal ischemia and bowel necrosis are rare complications of HVS and it is reported in newborn infants as necrotizing enterocolitis [7]. It is rarely reported in adults and we present an interesting case of bowel necrosis in a patient with newly diagnosed multiple myeloma thought to be due to HVS. 

Bowel necrosis is a surgical emergency defined by cellular death as a result of decreased blood flow to the gastrointestinal tract and can occur when the blood vessels supplying the bowel become occluded and prevent blood flow to the affected area. The most common reasons for bowel necrosis include acute arterial obstruction from thromboembolism with less common causes being venous thrombosis, HVS, etc. The exact mechanism by which HVS causes bowel necrosis is not entirely understood. However, it is thought that the thickened blood can cause increased pressure within the blood vessels, leading to damage and occlusion, relatively decreased microcirculation, and hypoperfusion of tissues. Also, the thickened blood may increase the risk of forming blood clots and further increase the risk of bowel necrosis [8].

Symptoms of bowel necrosis may include abdominal pain, constipation, bloating, fever, nausea, and vomiting. Patients will appear in distress and diaphoretic on physical exam. When there is a high degree of clinical suspicion, an evaluation must proceed quickly with an immediate CT scan of the abdomen pelvis with IV contrast along with initial hemodynamic support management. If the patient remains hemodynamically unstable in spite of adequate resuscitative measures, it is recommended that the patient be immediately taken to the operating room for emergency exploratory laparotomy. 

In patients with HVS, treatment mainly involves addressing the underlying condition causing the thickening of the blood. This may include chemotherapy, plasma exchange, or other interventions to reduce the viscosity of the blood to improve blood flow to the affected areas. When clinical suspicion is high for HVS, therapeutic apheresis should be considered to prevent complications and multiorgan failure [4,9]. A single session of plasma exchange can reduce plasma viscosity by 30-50% and immunoglobulin levels by 60% [9].

We believe that our patient represents an uncommon case of IgG MM associated with blood hyperviscosity and bowel necrosis. Early recognition and prompt treatment of HVS are essential to minimize the risk of complications and improve outcomes.

## Conclusions

Necrotizing enterocolitis can be a life-threatening condition and can be missed due to its complex presentation. It should be considered in the differential diagnosis of patients with abdominal pain, bloating, nausea, vomiting, or constipation. HVS is one of the uncommon complications of MM and could lead to bowel necrosis. Early recognition and prompt treatment are essential to minimize morbidity and mortality.
